# Relationship between perceived social support and psychological hardiness with family communication patterns and quality of life of oncology patients

**DOI:** 10.1002/nop2.808

**Published:** 2021-02-19

**Authors:** Fatemeh Haj Hashemi, Foroozan Atashzadeh‐Shoorideh, Parastoo Oujian, Bahram Mofid, Maryam Bazargan

**Affiliations:** ^1^ Student Research Committee, School of Nursing & Midwifery Shahid Beheshti University of Medical Sciences Tehran Iran; ^2^ Department of Psychiatric Nursing and Management School of Nursing & Midwifery Shahid Beheshti University of Medical Sciences Tehran Iran; ^3^ Department of Pediatric Nursing School of Nursing & Midwifery Shahid Beheshti University of Medical Sciences Tehran Iran; ^4^ Cancer Research Center Department of Radiation Oncology Shohada‐e‐Tajrish Medical Center School of Medicine Shahid Beheshti University of Medical Sciences Tehran Iran; ^5^ College of Nursing and Health Sciences Flinders University Adelaide SA Australia

**Keywords:** cancer, communication, nursing, oncology, patients, quality of life, resilience, social support

## Abstract

**Aim:**

The purpose of this study was to determine the relationship between PSS, PH, FCP and QoL of oncology patients.

**Methods:**

In this descriptive‐correlational study, 340 oncology patients were selected with convenience sampling method from the hospitals in Tehran 2018–2019. Data were collected using, “PSS,” “PH,” “FCP” and “European Organization for Research and Treatment of Cancer Quality of Life Questionnaire, EORTC QLQ‐C30.” Data were analysed using descriptive and inferential statistics using SPSS21 and Amos.

**Results:**

The direct effect and the total effect of PSS and FCP on QoL were significant (*p* < .001), but their indirect effect was not significant (*p* > .05) and the effect of PH on QoL was not significant (*p* = .96). The Root Mean Squares of Error Approximations (RMSEA), Non‐Normed Fit Index (NNFI), Comparative Fit Index (CFI) and Goodness of Fit Index (GFI) were estimated 0.07, 0.97, 0.98 and 0.91, respectively.

## 1 INTRODUCTION

Currently, cancer is one of the leading causes of mortality around the globe (Siegel et al., 2019). The data obtained from investigation of 36 types of cancer in 185 countries in 2018 demonstrated that 18 million people were affected with cancer, of whom 8.2 million died (DeSantis et al., 2019). Fear of cancer recurrence, anxiety about its complications and concerns with returning to normal life are among the challenges of oncology patients (Soriano et al., 2019).

There is extensive evidence on the importance of psychological care of oncology patients and their families (Sheikhzakaryaee et al., 2018). Studies have indicated that cancer diagnosis and treatment would lead to abundant somatic problems reduced life functions, familial disintegration (Henson et al., 2017), inefficient interpersonal relations, and disability in fulfilling social and familial responsibilities (O'Rourke & Lobchuk, 2018). Cancer affects many aspects of life and may lead to many temporary or permanent psychosocial problems. Therefore, integration of psychosocial aspects in management of oncology patients is necessary (Hagio et al., 2018).

QoL is one of the concepts that play a significant role in the field of life‐threatening disorders (Moshki et al., 2019). Some studies have suggested that QoL is influenced by many factors and that if these variables are improved, negative effects can be reduced and individuals’ QoL may be improved (Zhang et al., 2018). Perceived social support (PSS) is one of the important aspects of care for oncology patients (Kelley et al., 2019). Social support initiates with social contact, communication and safe support for the individual (Melissant et al., 2019). Effective social support helps individuals to cope with the stressful conditions and can lead to a better feeling towards themselves (Abadi Bavil & Dolatian, 2018). Studies conducted so far revealed that PSS plays a significant role in compatibility with refractory chronic conditions like cancer (Ozdemir & Tas Arslan, 2018).

Psychological hardiness (PH) including three components of control, commitment and challenge serves as a protection against stress, hindering the detrimental effects of stress on individuals’ health (Talavera‐Velasco et al., 2018). The results of one study have shown that QoL of a person can be predicted based on the level of their PH. Thus, it may be concluded that, similar to PSS, PH increases the QoL in oncology patients (Bahrami et al., 2018). Another variable influencing QoL is family communication pattern (FCP; Epstein et al., 2017). It is formed through interactions among family members and their beliefs and emotions (Ledbetter, 2019). FCP is assessable in the form of conversation orientations and conformity orientation. Conversation orientation provides some opportunities in which all family members can freely participate in familial discussions and debates where they deal with exchanging their thoughts and feelings about different patients. In conformity orientation, familial interactions aim at aligning beliefs, attitudes and values (Erdner & Wright, 2018).

## 2 BACKGROUND

It should be pointed out that some studies have measured the correlation between psychological hardiness and QoL among the female oncology patients (Bahrami et al., 2018) and healthy individuals (Talavera‐Velasco et al., 2018); nonetheless, more studies are required to identify the effect of this factor on QoL of oncology patients (Bahrami et al., 2018).

Due to the increase in the incidence of cancer and its effect on QoL, it is necessary to identify and improve the factors affecting promotion of QoL in oncology patients (Lehto et al., 2019). A cross‐sectional study conducted by Costa et al. on 144 patients with colorectal cancer indicated that social support by the family and other supporting resources decreased stress and increased QoL in patients (Costa et al., 2017).

Besides, very few studies have so far explored the familial communicative patterns of oncology patients (Epstein et al., 2017), and most studies have focused on healthy populations (Erdner & Wright, 2018; Ledbetter, 2019). Consequently, it appears that the three variables PSS, PH and FCP affect QoL separately; however, their simultaneous effect is unknown, yet. There are few studies about the relationship between perceived social support and psychological hardiness on oncology patients in Iran. Such studies are of particular interest in that the shift from institutional to home care is growing in Iran just as it is in Western countries.

### 2.1 Research aim

The aim of this study is to investigate the relationship between PSS, PH, FCP and QoL of oncology patients in oncology patients.

## 3 THE STUDY

### 3.1 Design

This descriptive‐correlational study was conducted in five public hospitals of Tehran City during 2018–2019.

### 3.2 Method

A total of 340 oncology patients were selected with convenience sampling method (Response rate = 91%). Inclusion criteria were a minimum of one‐month treatment, age of 18+ years and not being in the end stage of life. Uncompleted questionnaires were excluded. To determine the required sample size at confidence level of 95% with the correlation coefficient of 0.2, and error factor of 0.05 as well as the beta coefficient of 0.1, a sample size of 340 nurses was estimated using the related formula. Considering participants attrition rate of 10%, 370 oncology patients were selected for the study. Sixteen participants did not return the instruments, and 14 deficiently filled instruments were excluded; ultimately, 340 participants entered the study.

Data were collected via “demographic questionnaire,” “PSS,” “PH,” “FCP,” and “European Organization for Research and Treatment of Cancer Quality of Life Questionnaire, EORTC QLQ‐C30.”The demographic questionnaire included personal information.

"PS" has 12 items with three dimensions of “family,” “friends” and “other important family members” which was developed by Zimet et al. It uses a seven‐point Likert scale with the total score 12–84 (Zimet et al., 1988). The total Cronbach's *α* coefficient in the present study was 0.89.

The 50‐item PH questionnaire, developed by Kobasa, consisted of three subscales of commitment, challenge and control. It is based on the four‐point Likert scale with total score 0–150. The scale possessed an acceptable Cronbach's *α* coefficient (Kobasa, 1979). The total Cronbach's *α* coefficient of the inventory was 0.86 for all three subscales.

FCP questionnaire consists of two dimensions, has 26 items and was developed by Ritchie & Fitzpatrick (Fitzpatrick & Ritchie, 1994). It uses a five‐point Likert scale. The total score of this questionnaire is 42–108. The total Cronbach's *α* of the instrument obtained in this study was 0.89.

Moreover, EORTC QLQ‐C30 with three subscales of performance, symptoms and general status of life was applied; the validity of the Persian version of this questionnaire was approved by Montazeri. It is based on the four‐point Likert scale in items 1–28, and seven‐point Likert scale in items 29–30, with a total score of 0–104 (Montazeri et al., 1999). The Cronbach's *α* coefficient of the tool was 0.92 in the present study.

### 3.3 Analysis

Data were analysed using descriptive and inferential statistics with Amos and SPSS21. In the descriptive statistics, mean, standard deviation, frequency and percentage were reported. Pearson correlation coefficient was used to determine the relationship. To investigate the simultaneous relationship of main variables, regression analysis and the structural equation modelling was used.

### 3.4 Ethics

Ethical approval of the study was bestowed by Committee of Ethics in Human Research at SBMU with code of ethics no: IR.SBMU.RETECH.REC.1397.308. To observe the ethical principles, obtaining the informed written consent from the participants was done. In addition, the participants were made assured of the confidentiality and anonymity of information in the instruments.

## 4 RESULTS

The mean age of the patients was 43.99 ± 11.32 years, most of them (58.2%) were female, from urban areas (85%), married (83.8%), held a high school diploma (51.2%), unemployed (66.2%), affected with breast cancer (23.5%), Grade II (52.9%) and had a nuclear family structure (94.7%). The results of ANOVA and independent *t* tests showed that the mean score of QoL did not differ significantly between different groups (*p* >.05). (Tables 1 and 2).

**TABLE 1:** Demographic and work characteristics of the study participants

Variable	Category	*N* (%)	Mean (*SD*) quality of life	*p*‐value
Age (years)	20–30	45 (13.2)	75.67 (13.54)	.64*
	31–40	82 (24.1)	79.46 (14.75)	
	41–50	109 (32.1)	77.39 (15.31)	
	51–60	90 (26.5)	77.66 (14.95)	
	>60	14 (4.1)	75.07 (10.58)	
Gender	Male	198 (58.2)	78.18 (14.27)	.42**
	Female	142 (41.8)	76.87 (15. 23)	
Residence type	Village	51 (15.0)	76.55 (14.13)	.57**
	Urban	289 (85.0)	77.83 (14.78)	
Marriage status	Single	42 (12.4)	79.79 (13.72)	.39*
	Married	285 (83.8)	77.40 (14.86)	
	Divorced	4 (1.2)	84.25 (14.48)	
	Widowed	9 (2.6)	72.22 (12.77)	
Education	No degree	125 (36.8)	76.96 (13.84)	.86*
	Diploma	174 (51.2)	77.82 (15.51)	
	University degree	44 (12)	78.44 (13.52)	
Type of employment	Student	8 (2.4)	68.88 (14.13)	.15*
	Unemployed	225 (66.1)	76.71 (15.10)	.15*
	Non‐Governmental	82 (24.1)	79.68 (13.31)	
	Government Employee	25 (7.4)	81.24 (14.32)	
Number of children's	0–2	84 (24.7)	78.0 (14.65)	.57*
	3–4	170 (50)	77.5 (14.83)	
	>4	44 (12.9)	75.2 (15.22)	
	Single	42 (12.4)	79.79 (13.74)	
Type of cancer	Breast	80 (23.5)	76.4 (15.90)	.81*
	Reproduction system	38 (11.2)	78.5 (15.26)	
	Blood	79 (23.2)	78.6 (15.13)	
	Digestive system	75 (22.1)	76.3 (12.94)	
	Respiratory system	43 (12.6)	79.2 (12.84)	
	Other	25 (7.4)	78.4 (16.74)	
Grade	Grade 1	45 (13.3)	77.0 (15.28)	.88*
	Grade 2	180 (52.9)	77.5 (14.72)	
	Grade 3	155 (33.8)	78.2 (14.47)	
Family status	Nuclear	322 (94.7)	77.6 (14.61)	.84*
	Extended	5 (1.5)	80.4 (16.28)	
	Incomplete	13 (3.8)	75.9 (13.95)	

* ANOVA test.

** Independent *t* test.

**TABLE 2:** Value of research variables and their dimensions

Mean and standard deviation	Score range in scale	Highest score	Lowest score	Variables
17.79 (4.62)	28–4	28	5	Support from Family
16.34(4.13)	28–4	28	5	Support from Friends
15.46 (4.86)	28–4	27	5	Support from others
49.59 (7.95)	84–12	72	29	Perceived social support
23.97 (8.12)	48–0	46	3	Commitment
27.60 (9.19)	51–0	49	2	Challenge
25.49 (8.52)	51–0	47	2	Control
77.07 (16.03)	150–0	121	40	Psychological hardiness
29.14 (10.52)	60–0	54	1	Conversation
20.73 (8.24)	44–0	43	1	Conformity
49.86 (6.99)	104–0	75	24	Family communication Pattern
12.78 (2.54)	20–5	19	5	Function
64.86 (13.29)	92–23	90	25	Symptoms
7.35 (2.17)	14–2	14	2	General status
77.64 (14.67)	126–30	108	42	Quality of life

The direct effect and the total effect of PSS and FCP on QoL were significant, but their indirect effect was not significant and the effect of PH on QoL was not significant (Table 3).

**TABLE 3:** Direct and indirect effects of PSS, PH and FCP on QoL

Variable	Direct effect	*p*‐value	Indirect effect	*p*‐value	Total effect	*p*‐value
PSS	0.72	<.001	0.08	.12	0.8	<.001
PH	0.03	.96	0		0.03	.96
FCP	−0.54	<.001	−0.001	.98	−0.54	<.001

**FIGURE 1:**
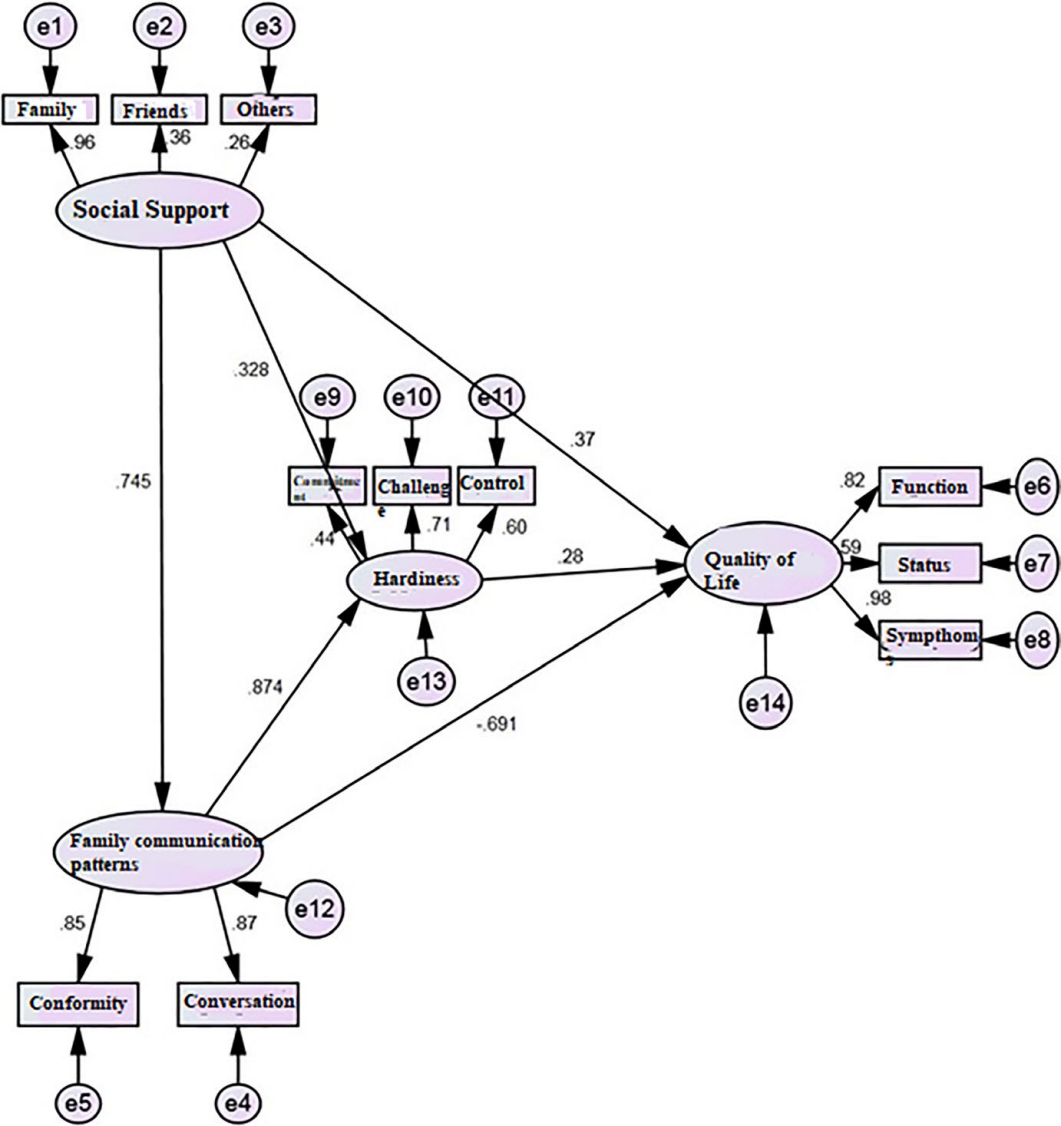
Fitted model with standard coefficients of perceived social support, psychological hardiness and family communication patterns with quality of life of oncology patients

The Root Mean Squares of Error Approximations (RMSEA), Non‐Normed Fit Index (NNFI), Comparative Fit Index (CFI) and Goodness of Fit Index (GFI) were estimated 0.07, 0.97, 0.98 and 0.91, respectively. RMSEA values less than 0.10 and CFI, GFI and NNFI values greater than 0.90 indicate good fit of the model. Figure 1 shows the fitted model with standard coefficients.

Figure 1

## 5 DISCUSSION

The aim of this study was to determine the relationships between “PSS,” “PH,” and “FCP” with “QoL” in oncology patients. The result of correlation between PSS, FCP, PH and QoL showed a significant positive correlation. However, FCP showed a negative correlation with PH and QoL. Finally, PH showed a significant positive correlation with QoL. In one study on the correlation between PSS, PH and FCP with QoL, the authors concluded that FCP was significantly correlated with PH and QoL of patients (Souri & Ashoori, 2015). Moreover, one study showed a positive significant correlation between PSS with FCP (High & Scharp, 2015). Besides, NG et al. demonstrated in their study on oncology patients that PSS was an important factor in enjoying a higher QoL (Ng et al., 2015). Li et al. showed that any attempt made to enhance social support, hope and resilience can increase QoL (Li et al., 2016). This is consistent with our finding that the greater the PSS of the target group the better their QoL was.

Furthermore, Senneseth et al. suggested that there was a reverse correlation between hardiness and social support with QoL (Senneseth et al., 2017). PH consists of a mixture of beliefs about the self and the world and includes components of commitment, control and challenge (Jeong et al., 2016). A person with high responsibility is aware of the value and importance of self in performing daily activities. A person with a sense of control feels effective in their course of life. Finally, someone who possesses the subscale of “challenge,” looks at life events as an opportunity for growth and development (Tsai & Lu, 2018). Thus, people with psychological hardiness have a high adaptability to environmental and psychological pressures and, unlike others, evaluate stressful events more positively and controllably and choose more effective coping methods. As a result, they experience less negative consequences of stress then have a higher quality of life.

The results of study by Tsai & Lu conducted in 2018 on cancer survivors demonstrated that PSS was significantly correlated with QoL (Tsai & Lu, 2018). Moreover, Dura et al. concluded in their study on elderly cancer survivors that personality and social support affect the elongation of feeling of well‐being and betterment of QoL in the target group (Durá‐Ferrandis et al., 2017). The results of the study by Ng CG et al. on breast cancer patients indicated that PSS was an important factor in improving QoL and decreasing mental pressure. This highlights the importance of the activities that promote and maintain the social support system for breast cancer patients (Ng et al., 2015).

The study by Pfaendler KS demonstrated that provision of supportive care during treatment and assessment of the effects of supportive care could diminish the prevalence and magnitude of long‐term complications of cancer of cervix, finally leading to improved QoL and quality care (Pfaendler et al., 2015).

Additionally, Pandey et al. found in their study entitled: “The effect of the mediating role of social support on the correlation between hardiness and immune response” that if social support exists in the model, the total effect of psychological hardiness on the immune response will be decreased (Pandey & Shrivastava, 2017).

Weiss et al. suggested that the greater the rate of perceived social support and self‐efficacy in parents with autistic children, the higher their psychological hardiness will be, resulting in their reduced anxiety on responding to stressors (Weiss et al., 2013).

Interpreting this finding, we can say that social support can be like a shield against stressful events that people experience. Social support can also reduce isolation and create a sense of worth, thus improving the quality of life. The findings of our study showed that FCP has a significant negative correlation with QoL. Sanavi et al. showed in their study on teenagers that FCP was significantly positively correlated with QoL (Sanavi et al., 2013). This is not consistent with our findings. This may be attributed to differences in the study populations. The correlation between FCP and QoL varies among healthy and unhealthy individuals, especially oncology patients. Since QoL is influenced by numerous factors, such a finding was expected in the present study. It should be pointed out that FCP can serve as a shield against overwhelming events and accidents experienced by individuals during their life span (High & Scharp, 2015). The more the individuals manifest their emotions and excitements in the family environment, the more they are encouraged to betray their feelings; also, the more openly they discuss their affairs, the greater QoL they will enjoy (Sanavi et al., 2013). Furthermore, the reinforcement of familial communication would predispose to the use of efficient conflict resolution methods, healthy relations, increased self‐esteem, stronger interpersonal communicative skills, higher tolerance and resilience, and developed independence.

Finally, Costa et al. claimed that social support, especially familial support, is an important factor in promoting QoL in these individuals (Kelley et al., 2019). The results of our study showed good fit of SEM model of the significant correlations between PSS, PH and FCP with QoL.

One strong point of the present study was the fitting of the conceptual model that, in line with healthcare interventions, may aid in improving PH, PSS and FCP by the healthcare team to enhance the patients’ QoL. The role of family members in patient care is reinforced because of social structures, including family bonds in Eastern countries (Effendy et al., 2015). Cultural differences relate to beliefs about a patient's death and the ethical and cultural challenges related to how oncology staffs discuss a cancer diagnosis with patients (Kazdaglis et al., 2010). In the Middle East, a cancer diagnosis is accompanied by social stigma. In Middle Eastern countries, including Iran, the majority of populations are Muslim and social structures are based on family cohesion and patient–family relationship (Khalil, 2013). Therefore, considering the close relationship between family members in Iran and the transfer of a large proportion of patient caregivers at home, it is important to pay attention to the relationship between the perceived social support and psychological hardiness and quality of life of this study.

## 6 LIMITATIONS

There was one limitation in this study. Some potential associated factors of PH, PSS, FCP and QoL such as type of chemotherapy drugs, religion, understanding and knowledge of the illness were not measured.

## 7 CONCLUSION

According to the structural model of the study, it can be concluded that nurses ought to pay attention to the signs and symptoms of these variables and, on this basis, develop some suitable programs to improve QoL in oncology patients. The results of the present study can increase the importance of the nursing knowledge in recognizing the importance of PSS, PH and FCP in patients to improve QoL of the patients. Giant strands may be taken to promote health and QoL of patients through identifying the factors that affect the variables under study and providing the necessary related training to the treatment team, especially nurses due to the importance of PSS, PH and FCP in QoL of oncology patients. These factors exert some effects on patients’ motivation and inclination for adherence to treatment.

## CONFLICT OF INTERESTS

The authors declare that they have no competing interests.

## AUTHOR CONTRIBUTIONS

All authors (FH, FA, PO, BM and MB) have participated in the conception and design, analysis and interpretation of data, read and approved the final manuscript. FH: Data collection. FH, FA, PO, BM and MB: Drafting the manuscript or revising it critically for important intellectual content.

## Data Availability

The data that support the findings of this study are available on request from the corresponding author. The data are not publicly available due to privacy or ethical restrictions.
